# Molecular Epidemiology of Human Enterovirus Associated with Aseptic Meningitis in Shandong Province, China, 2006–2012

**DOI:** 10.1371/journal.pone.0089766

**Published:** 2014-02-21

**Authors:** Zexin Tao, Haiyan Wang, Yan Li, Guifang Liu, Aiqiang Xu, Xiaojuan Lin, Lizhi Song, Feng Ji, Suting Wang, Ning Cui, Yanyan Song

**Affiliations:** 1 Academy of Preventive Medicine, Shandong University, Jinan, People’s Republic of China; 2 Shandong Provincial Key Laboratory of Infectious Disease Control and Prevention, Shandong Center for Disease Control and Prevention, Jinan, People’s Republic of China; 3 School of Public Health, Shandong University, Jinan, People’s Republic of China; 4 Department of Preventive Medicine, College of Basic Medical Sciences, Shandong University of Traditional Chinese Medicine, Jinan, People’s Republic of China; Johns Hopkins School of Public Health, United States of America

## Abstract

**Background:**

Human enteroviruses (HEVs) are common causes of acute meningitis. However, there is limited information about HEV associated with aseptic meningitis in mainland China because it has not been classified as a notifiable disease.

**Objectives:**

To characterize the HEVs associated with sporadic aseptic meningitis in China and to analyze their genetic features.

**Study Design:**

Cerebrospinal fluid, throat swab and feces specimens were collected from patients with aseptic meningitis in 5 sentinel hospitals in Shandong Province, China between 2006 and 2012. Virological investigation (viral isolation and molecular identification) and phylogenetic analysis were performed.

**Results:**

A total of 437 hospitalized patients were reported, and enteroviruses were detected in the specimens from 84 patients (19.2%) and were identified into 17 serotypes. The nine main serotypes were echovirus (E) 30 (27.4%), EV71 (13.1%), coxsackievirus (CV) B1 (9.5%), CVB3 (7.1%), CVB5 (7.1%), E6 (7.1%), E9 (7.1%), CVA9 (6.0%), and CVA10 (3.6%). Monthly distribution of isolated enteroviruses revealed a major peak in summer-fall season and a small second peak in winter constituted totally by EV71. Sequence analysis on VP1 coding region suggested Shandong strains had great genetic divergence with isolates from other countries.

**Conclusions:**

Multiple serotypes were responsible for enterovirus meningitis in mainland China. Aseptic meningitis caused by EV71 and coxsackie A viruses–the predominant pathogens for the hand, foot, and mouth disease–is currently an important concern in mainland China.

## Introduction

Human Enteroviruses (HEVs) belong to family *Picornaviridae*. They are common pathogens associated with various clinical syndromes, from minor febrile illness to severe, potentially fatal diseases such as aseptic meningitis, encephalitis, paralysis, myocarditis, and neonatal enteroviral sepsis [1]. HEVs are the major causative agents of aseptic meningitis in many parts of the world, and numerous HEV associated aseptic meningitis epidemics and outbreaks have been described [1], [2].

In China, several investigation on HEV associated aseptic meningitis outbreaks have been reported, such as echovirus (E) 30 in Jiangsu Province in 2003 [3], E6 in Anhui in 2005 [4], coxsackievirus (CV) A9 in Gansu in 2005 [5], E30, CVB3 and CVB5 in Shandong in 2003, 2008 and 2009, respectively [6]–[8]. These investigations were triggered by the huge number of hospitalized children and the attention of public health officials, not by surveillance data because aseptic meningitis has not been classified as a notifiable disease in China, and there has been to date no specific enterovirus surveillance system. So, the information about the circulating HEV causing aseptic meningitis in the population of China is limited.

Shandong is a coastal province with a population of 95.79 million (2010 census data). To investigate the serotypes and molecular epidemiological characterization of HEV associated with meningitis, a prospective surveillance on aseptic meningitis was conducted in 5 sentinel hospitals in Shandong Province from 2006 to 2012. Cerebrospinal fluid (CSF) was the main specimen, and throat swab and stool specimens were also collected. Virus isolation and molecular epidemiology of the isolates was performed. The epidemic pattern of HEV, along with the clinical severity associated with some serotypes was also analyzed.

## Materials and Methods

### Patients and Specimens

Shandong Province is located in the eastern part of China with an area of 156,700 km^2^. Jinan is the capital city, and Linyi is the largest city in Shandong, with total populations of 6.8 million and 10.0 million, respectively. Aseptic meningitis cases <15 years of age admitted to 4 sentinel hospitals in Jinan city from 2006 to 2012 and 1 sentinel hospital in Linyi city from May 2010 to Jun 2011 were studied. All meningitis patients were diagnosed by clinical doctors in the local hospital, in accordance with the diagnostic criteria referenced by Mirand et al. [9]. CSF, throat swab and stool specimens were collected at the time of admission, maintained at about 4°C during sample transport, and stored at −20°C.

The ethical approval was given by Ethics Review Committee of the Shandong Center for Disease Control and Prevention, and the study was conducted in compliance with the principles of the Declaration of Helsinki. Written informed consents for the use of their clinical samples were obtained from the parents or legal guardians of the patients.

### Virus Isolation and Serotyping

The stool specimens were processed according to standard protocols for poliovirus isolation recommended by WHO [10]. The throat swab specimens were shacked and filtered through a 0.22-µm-pore-size filter. Cerebrospinal fluid specimens were inoculated directly without treatment. RD and HEp-2 cell lines were used for virus isolation. Both cell lines were gifts from the WHO Global Poliovirus Specialized Laboratory in USA and were originally purchased from the American Type Culture Collection (ATCC). A total of 200 µl of treated solution was added to each of the cell culture tubes. After inoculation, the tubes were kept in a 36°C incubator and were examined daily. After 7 days, the tubes were frozen and thawed and repassaged, and another 7-day examination was performed. In order to prevent cross contamination, cell tubes of normal RD and HEp-2 cells served as negative controls. When cytopathic effect (CPE) was observed, microneutralization assays were carried out in 96-well tissue culture plates using antibody pools A to H for enterovirus (National Institute for Public Health and the Environment, [RIVM], Netherlands). Briefly, the antiserum-virus mixtures were incubated for 1 h at 36°C. Subsequently, 100 µl of suspension fluid of RD or HEp-2 cells was added to the plate, which was subsequently examined daily for the presence of CPE. The antiserum that inhibited the development of CPE was evaluated according to the manufacturer’s instructions.

### VP1 Amplification and Sequencing

For RT-PCR amplification, no passage is conducted and total RNA was extracted directly from 140 µl of virus stocks using a QIAamp viral RNA mini kit (Qiagen, Valencia, CA) according to the manufacturer’s procedure. Reverse transcription-PCR (RT-PCR) was performed using an Access RT-PCR System (Promega). Different primer pairs (486/488 and 040/011 for HEV-A, 008/013 and 187/011 for HEV-B) were used to amplify partial VP1 coding region, and the combination of two sequences yielded the entire VP1 coding region [11]–[13]. In order to prevent cross-contamination, an RT-PCR using the RNA extracted from normal RD cells served as a blank control, and a negative control containing all the components of the reaction mixture except for the template was also included.

The products were analyzed by agarose gel electrophoresis, and positive products were purified and sequenced directly with a BigDye Terminator v3.1 Cycle Sequencing Kit (Applied Biosystems, Foster City, CA). Sequences were analyzed by an ABI 3130 genetic analyzer (Applied Biosystems). Molecular typing was performed using online Enterovirus Genotyping Tool version 0.1 [14].

### Sequence Analysis

Nucleotide sequence alignments were carried out by BioEdit 7.0.5.3 software [15]. Phylogenetic trees were constructed by Mega 4.0 [16] using neighbor-joining method after estimation of genetic distance using the Kimura two-parameter method [17]. A bootstrapping test was performed with 1,000 duplicates, and the transition/transversion rate was set at 2.0.

### Nucleotide Accession Numbers

VP1 sequences determined in this study were deposited in GenBank under accession numbers GQ246517–GQ246520, GQ329744, GQ329747, GQ329838, HQ829953, JQ364849, JQ364850, JF823636–JF823646, JX138493–JX138496, KF150144–KF150174, and KF246747–KF246774.

## Results

### Cases and Epidemiology

From 2006 to 2012, a total of 437 aseptic meningitis cases (326 from Jinan and 111 from Linyi) were reported from the sentinel hospitals. Age of cases ranged from 4 months to 12 years old, and most (83.8%) were <5 years of age. The gender ratio was 2.83∶1, with 323 male and 114 female cases, suggesting greater exposure of male children.

For all cases investigated, fever (94.5%), vomiting (55.8%) and headache (42.3%) were the most common clinical manifestations, and others included lethargy (20.8%), convulsion (11.7%), listlessness (11.7%), diarrhea (10.8%), and unconsciousness (7.1%). No sequel or death was reported. For HEV positive cases, fever (97.6%), vomiting (60.7%) and headache (48.8%) were the most common clinical manifestations, and others included listlessness (11.9%), lethargy (9.5%), convulsion (4.8%), diarrhea (4.8%), and unconsciousness (2.4%). The clinical information of EV71 and species A CVs associated patients is shown in Table 1.

**Table 1:** Information of EV71 and species A coxsackieviruses isolated from aseptic meningitis patients in Shandong, China in 2006–2012.

Strain	Serotype	GenBank no.	Patient		
			Gender	Age	Clinical symptom
049/LY/CHN/10	EV71	KF150164	F	2 y	Fever, vomiting, lethargy
084/LY/CHN/10	EV71	KF150165	M	1 y	Fever, vomiting, convulsion
085/LY/CHN/10	EV71	KF150166	M	2 y	Fever, vomiting, headache
102/LY/CHN/10	EV71	KF150167	M	1 y	Fever, vomiting, convulsion
103/LY/CHN/10	EV71	KF150168	M	3 y	Fever, vomiting, headache
120/LY/CHN/10	EV71	KF150169	M	3 y	Fever, vomiting, headache, lethargy
123/LY/CHN/10	EV71	KF150170	M	4 y	Fever, vomiting, headache, lethargy
127/LY/CHN/10	EV71	KF150171	F	2 y	Fever, unconsciousness
128/LY/CHN/10	EV71	KF150172	M	1 y	Fever, lethargy
201/LY/CHN/10	EV71	KF150173	M	1 y	Fever, vomiting, listlessness
202/LY/CHN/11	EV71	KF150174	F	1 y	Fever, vomiting, listlessness
004/LY/CHN/10	CVA4	KF150144	F	1 y	Fever, lethargy
001/LY/CHN/10	CVA10	KF150147	F	2 y	Fever, vomiting, convulsion
016/LY/CHN/10	CVA10	KF150148	F	2 y	Fever, vomiting, convulsion
031/LY/CHN/10	CVA10	KF150149	M	2 y	Fever, vomiting, headache
048/LY/CHN/10	CVA16	KF150150	M	2 y	Fever, vomiting, headache

### Virus Isolation and Typing

Eighty-four enteroviruses (42 in Jinan and 42 in Linyi) and 3 human adenoviruses were isolated. Thirty-eight enteroviruses were isolated only in RD cell, 13 were isolated only in HEp-2 cell, and the rest 33 isolates were isolated in both cell lines. Serotyping was performed on all isolates, and serotypes of E30, E6, E9, CVA9, E3, E4, E25 and CVB were revealed from 65 isolates, while the other 19 isolates could not be typed by serological method. VP1 amplification was further performed on all 84 isolates, and all have positive results. Further VP1 sequencing and molecular typing revealed a total of 17 different HEV types. E30 was identified in 23 of these isolates (27.4%). Others included EV71 (13.1%), CVB1 (9.5%), CVB3 (7.1%), CVB5 (7.1%), E6 (7.1%), E9 (7.1%), CVA9 (6.0%), CVA10 (3.6%), CVB4 (2.4%), E24 (2.4%), CVA4 (1.2%), CVA16 (1.2%), E25 (1.2%), E3 (1.2%), E4 (1.2%), and E16 (1.2%) (Table 2). The 19 isolates that could not be typed serologically were EV71, CVA4, CVA10, CVA16, E16, and E24, respectively. Table 2 shows the results of annual isolation. Because the surveillance in Linyi was conducted in 2010–2011, the isolation number in these two years was relative higher.

**Table 2:** Isolation and homologous comparison of human enteroviruses from Shandong meningitis patients.

Serotype	Annual No. of isolates								% VP1 nt identity	
	06	07	08	09	10	11	12	Sum (% of total detected)	with each other	with prototype strain*
HEV-A										
CVA4	0	0	0	0	1	0	0	1 (1.2)	/	85.3
CVA10	0	0	0	0	3	0	0	3 (3.6)	99.4–99.6	76.6–76.7
CVA16	0	0	0	0	1	0	0	1 (1.2)	/	75.7
EV71	0	0	0	0	10	1	0	11 (13.1)	97.9–99.8	81.7–82.1
Total	0	0	0	0	15	1	0	***16 (19.0)***		
HEV-B										
CVA9	1	0	1	0	3	0	0	5 (6.0)	92.3–95.5	79.9–81.6
CVB1	0	0	0	0	0	8	0	8 (9.5)	97.6–99.7	78.7–79.2
CVB3	2	2	1	0	0	0	1	6 (7.1)	92.0–99.6	78.9–80.3
CVB4	0	0	0	0	2	0	0	2 (2.4)	98.0	82.7–83.2
CVB5	0	6	0	0	0	0	0	6 (7.1)	95.0–99.7	78.7–79.3
E3	0	0	0	0	0	0	1	1 (1.2)	/	80.6
E4	0	0	1	0	0	0	0	1 (1.2)	/	81.8
E6	0	2	0	0	3	1	0	6 (7.1)	78.5–99.8	77.2–79.3
E9	0	0	3	0	3	0	0	6 (7.1)	91.9–100.0	78.7–79.3
E16	0	0	0	0	1	0	0	1 (1.2)	/	81.2
E24	2	0	0	0	0	0	0	2 (2.4)	99.6	80.7–80.9
E25	0	1	0	0	0	0	0	1 (1.2)	/	80.9
E30	1	1	3	1	14	2	1	23 (27.4)	83.7–100.0	80.5–83.4
Total	6	12	9	1	26	11	3	***68 (81.0)***		

*The prototype strains (with GenBank accession number) were: CVA4-High Point (AY421762), CVA10-Kowalik (AY421767), CVA16-G10 (U05876), EV71-BrCr (U22521), CVA9-Grigg (D00627), CVB1-conn-5 (M16560), CVB3-Nancy (M16572), CVB4-E2 (S76772), CVB5-1954/UK/85 (X67706), E3-Morrisey (AY302553), E4-Pesacek (AY302557), E6-Charles (U16283), E9-Hill (X84981), E16-Harrington (AY302542), E24-DeCamp (AY302548), E25-JV-4 (AY302549), and E30-Bastianni (AF162711).

**Figure 1:**
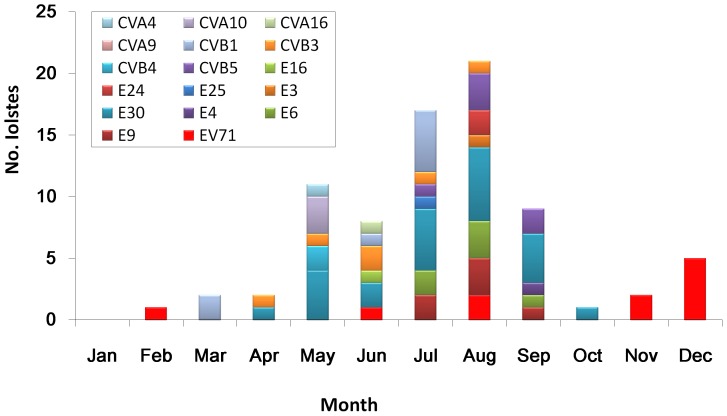
Monthly distribution of different serotypes isolated from aseptic meningitis cases in Shandong, 2006–2012. E, echovirus; CV, coxsackievirus; EV, enterovirus.

The monthly distribution of different serotypes is illustrated in Fig. 1. Most HEVs were isolated in summer season when most cases were reported. However, a minor peak of EV71 was observed during this study in December. As the most frequently isolated serotype, E30 was observed from April to October in the study period.

### Phylogenetic Analysis and Homologous Comparison

The complete VP1 sequences of the isolates from viral meningitis were aligned with global reference sequences and those previously obtained from cases with acute flaccid paralysis (AFP), HFMD and aseptic meningitis in Shandong Province.

**Figure 2:**
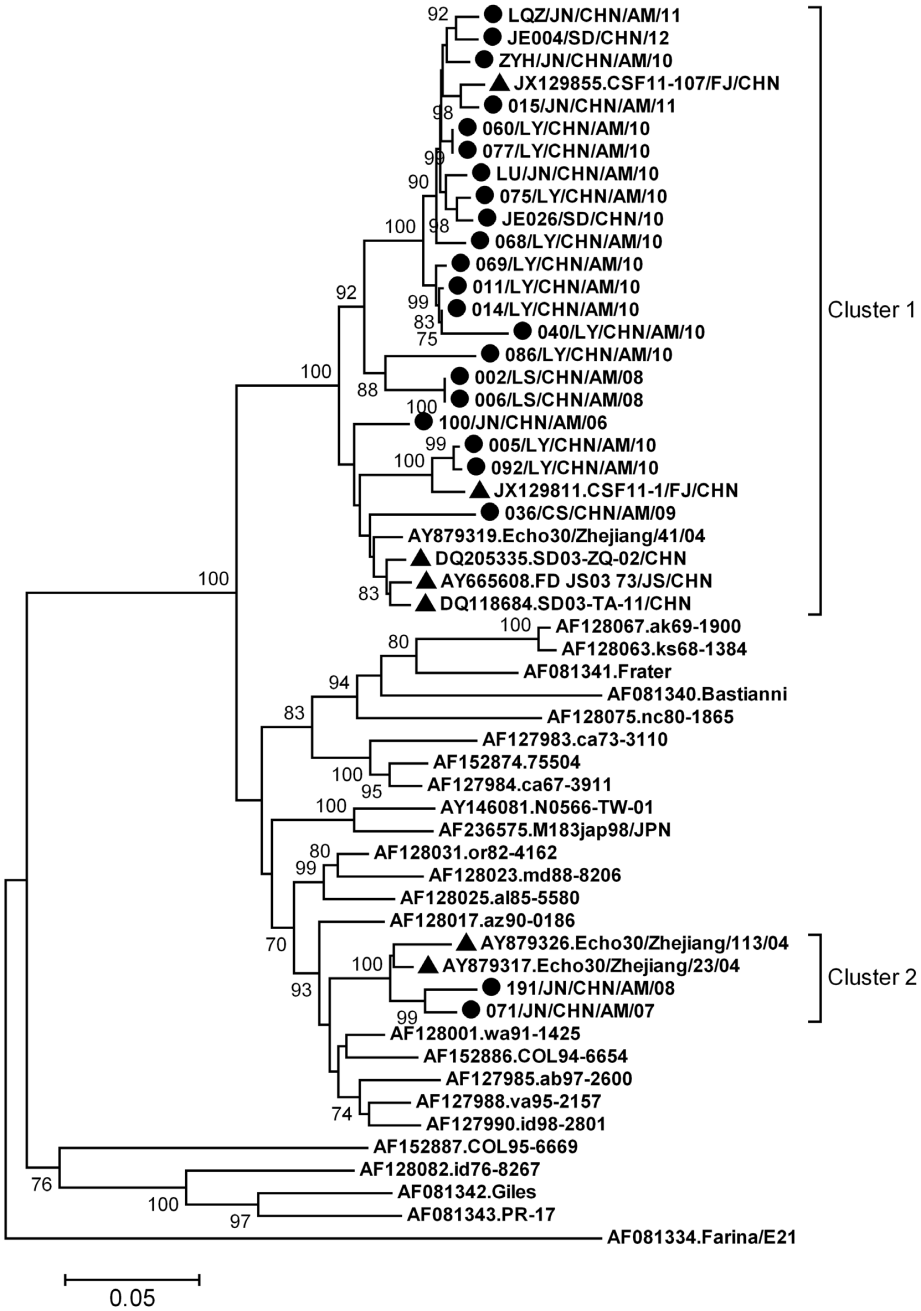
Phylogenetic tree based on E30 VP1 sequences of Shandong isolates and global strains. Chinese strains are grouped into two clusters. Circles indicate strains obtained in this study, triangles indicate reference strains from previously reported aseptic meningitis outbreaks in the provinces of Zhejiang, Jiangsu, Fujian and Shandong of mainland China.

Phylogenetic analysis showed Chinese E30 strains were clustered into two clusters (Fig. 2). Most Shandong isolates belonged to cluster 1, which contained most of the other domestic reference strains from aseptic meningitis outbreaks in the provinces of Shandong, Jiangsu, Fujian and Zhejiang, suggesting this predominant cluster is responsible for most outbreaks and sporadic cases of E30 associated aseptic meningitis in mainland China. E30 isolates from Shandong meningitis patients had 89.8% to 100.0% VP1 nucleotide identities among themselves, and 91.2% to 95.4% VP1 similarities with other domestic strains of this cluster. Cluster 2 was a minor one with only 4 isolates from Shandong and Zhejiang Province. They had 94.8% to 97.2% VP1 similarities with each other. Chinese strains in cluster 1 and cluster 2 had 75.1% to 96.0% and 75.1% to 91.3% VP1 similarities with foreign strains, respectively.

**Figure 3:**
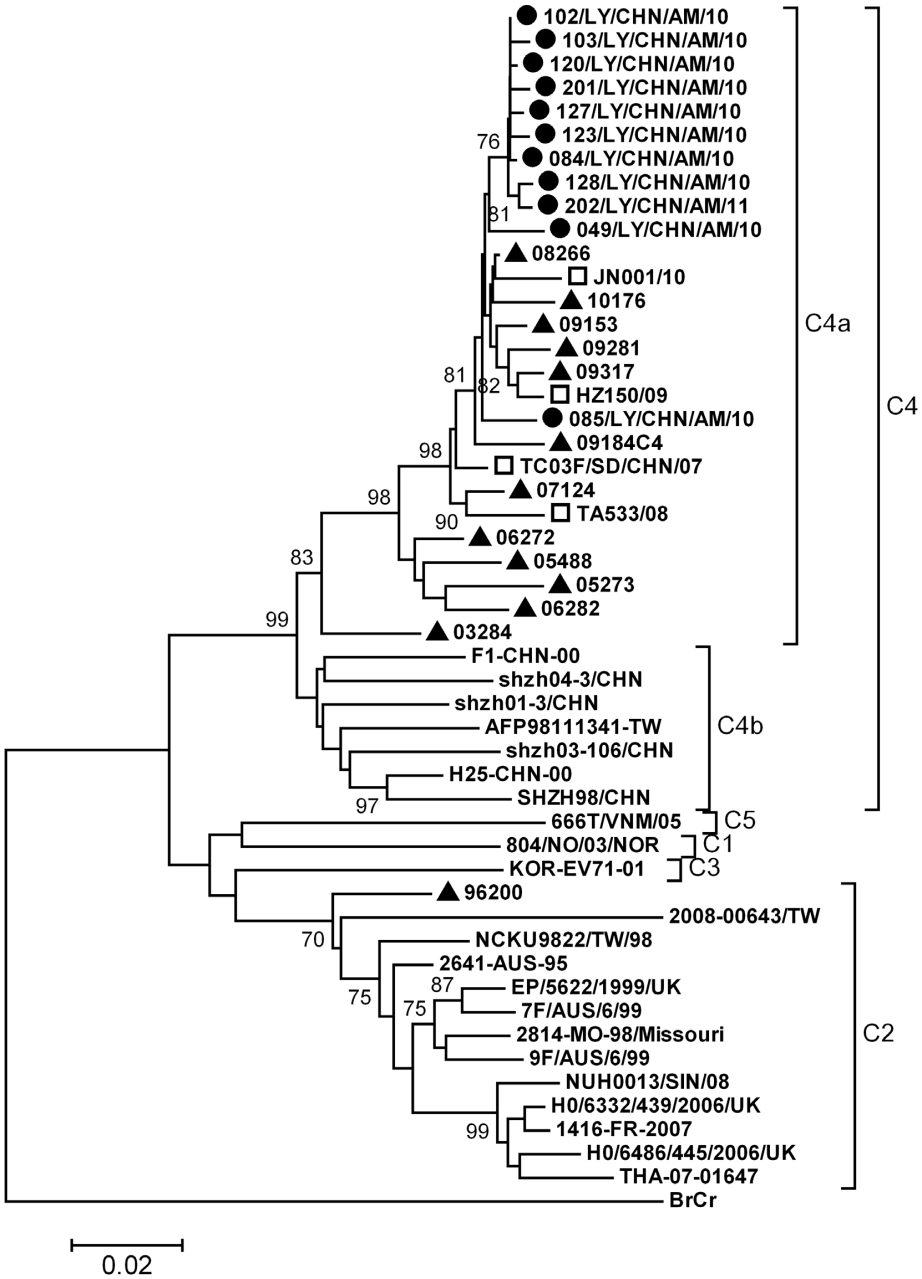
Phylogenetic tree based on EV71 VP1 sequences of Shandong isolates and reference strains of genogroup C. EV71 Strains from Shandong meningitis patients belong to C4a subgenogroup. Circles indicate strains obtained in this study, triangles indicate Shandong AFP strains, and squares indicate Shandong HFMD reference strains in 2007 to 2010.

EV71 isolates from Shandong meningitis patients had 97.9% to 99.8% VP1 nucleotide sequence similarities among themselves, and the mean *p*-distances is 0.009. Previous study revealed all EV71 isolates could be grouped into genogroups A, B, and C and corresponding subgenogroups. Subgenogroup C2, C3, C4 and C5 have been identified in mainland China [18]–[21], and C4 is the predominant subgenogroup responsible for most documented HFMD outbreaks and epidemic since 1998 [20]. Shandong strains from meningitis patients belonged to subgenogroup C4a (Fig. 3). They were closely related to local HFMD and AFP isolates collected from 2007 to 2010. These results suggest that since the large-scale epidemics of HFMD from 2007, EV71 associated serious neurological diseases have become a major public health concern in mainland China.

**Figure 4:**
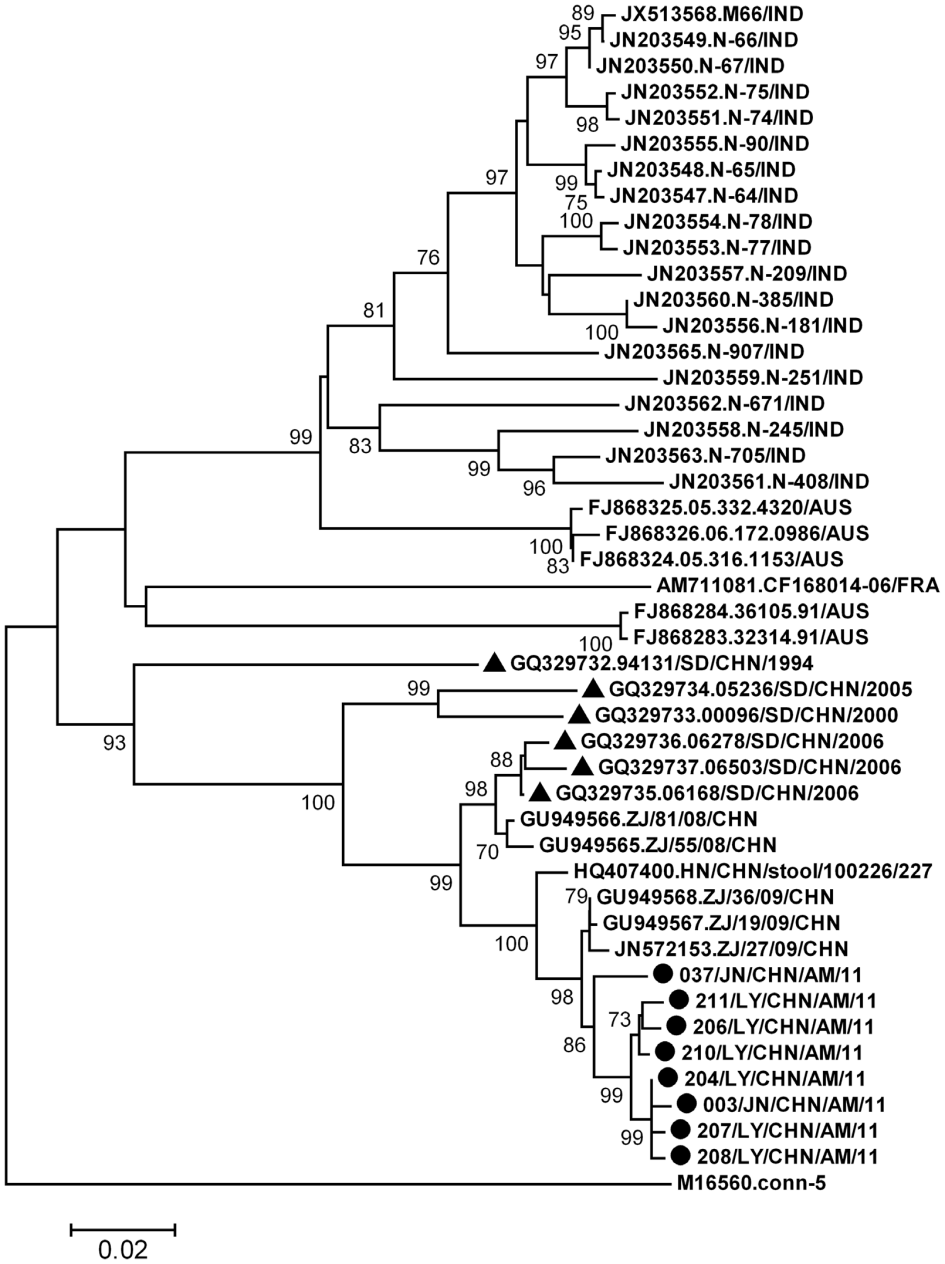
Phylogenetic tree based on CVB1 VP1 sequences of Shandong isolates and global strains. Circles indicate strains obtained in this study, triangles indicate Shandong AFP strains.

CVB1 isolates from Shandong meningitis patients had 97.6% to 99.7% VP1 nucleotide sequence similarities among themselves. Phylogenetic analysis revealed global CVB1 were grouped into 2 clusters. All Chinese strains from the provinces of Shandong, Zhejiang and Henan formed an exclusive cluster in which Shandong isolates from aseptic meningitis were segregated into a single lineage of this cluster (Fig. 4). Another cluster comprised of stains mainly from AFP and acute diarrhea patients in India and Australia [22], [23].

**Figure 5:**
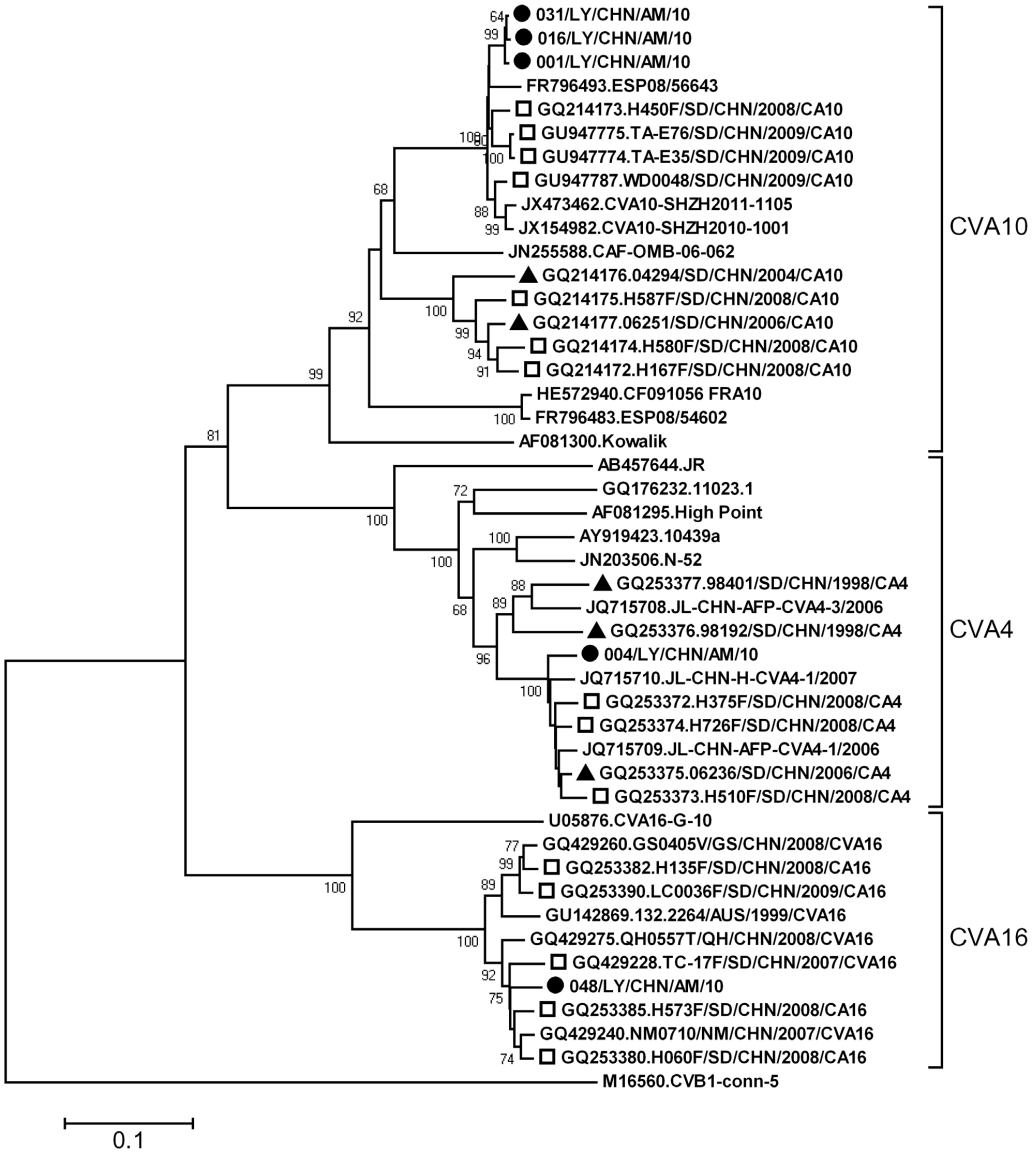
Phylogenetic tree based on VP1 sequences of CVA4, CVA10 and CVA16 among Shandong isolates and reference strains. Circles indicate strains obtained in this study, triangles indicate Shandong AFP strains, and squares indicate Shandong HFMD strains.

CVA10, CVA4 and CVA16 have been demonstrated to be the causative agents of HFMD [24]–[26]. In this study, they were also detected from patients with aseptic meningitis, although they accounted for a small proportion of reported cases. Phylogenetic analysis showed they were closely related to the local HFMD isolates of each serotype (Fig. 5).

## Discussion

The present study describes the serotypes and molecular epidemiology of human enteroviruses associated with aseptic meningitis in Shandong Province, China from 2006 to 2012. In USA, the National Enterovirus Surveillance System (NESS) has provided valuable information for investigating temporal patterns of circulation of different serotypes, guiding outbreak investigations, and identifying targets for development of diagnostic assays and antivirals [1], [27]. Whereas in China, HEV surveillance based on human specimens is very limited and mainly includes testing of specimens collected through AFP surveillance and HFMD surveillance. This study represents the first effort to conduct prospective surveillance for HEV associated meningitis in mainland China.

As no single cell line is capable of growing all human enteroviruses, it is common practice to use several types of cells to increase the spectrum of viruses that can be detected. Two cell lines were used in this study. HEV-A strains (such as EV71 and CVAs) can only be isolated and propagated in RD cell, and CVBs were mostly isolated via HEp-2 cell. The different viral receptor is supposed to be the cause of the cell tropism of different HEVs.

Compared with the results of serotype distribution from similar studies in other countries such as India, Brazil, and France [9], [28], [29], the most distinctive finding of this study is the observation of EV71 and other coxsackie A viruses in meningitis patients. In mainland China, large-scale outbreaks of HFMD have occurred repeatedly since 2007. EV71 and CVA16 are the two major causative agents in different areas, and other coxsackieviruses of species A, such as CVA10, CVA6, CVA4, CVA12, etc, have also been demonstrated to be involved [24]–[26]. Previous studies demonstrated EV71 can cause severe complications including aseptic meningitis, encephalitis and poliomyelitis-like paralysis [30]–[32], and CVA10 is also associated with serious complications with a less effect than EV71 [24]. In this study, EV71, CVA10, CVA4 and CVA16 accounted for 19.0% of overall detections. Sequence analysis revealed they were closely related to local HFMD isolates. These results suggest that aseptic meningitis caused by these HFMD agents is an important concern in mainland China in the context of repeatedly HFMD epidemic in mainland China since 2007. However, since this is the first effort to conduct prospective surveillance for HEV associated meningitis in mainland China, we could not obtain previous information on meningitis caused by EV71 and other CVAs. Further long-term case-based surveillance will provide more valuable data for understanding the circulation and molecular epidemiology of HEVs causing meningitis.

Moreover, in a recent study, CVA9, CVB1, CVB4, E6, E25 and E30 have been isolated in severe HFMD patients, although they accounted for a very small proportion [33]. These serotypes have also been demonstrated to be accounted for a majority of aseptic meningitis patients of this study. So, although the main serotypes of causative agents for HFMD and aseptic meningitis in mainland China is quite different (EV71 and species A CVs for HFMD, while echoviruses and CVBs for meningitis), the specification for these two groups of HEV in the pathogenicity is not completely clear and overlaps do exist.

Our study revealed that most enteroviruses were detected in summer-fall season, consistent with the seasonality of HEV infections in temperate climates [1]. Previous study showed that epicurves of number of weekly reported HFMD cases in mainland China are characterized by a major peak in summer season followed by a small second peak in early winter, and the re-opening of schools after the summer break is speculated to partially account for the second rise after September [34]. Consistently, in this study, a minor peak of EV71 was also observed in December (Fig. 1), suggesting high EV71 activity at that time accounted for a proportion of HFMD and meningitis patients.

E30 is one of the most frequently isolated serotype in many regions of the world and has caused many large outbreaks of aseptic meningitis in the last 40 years [2]. In China, the documented E30 associated aseptic meningitis outbreak occurred in the provinces of Shandong and Jiangsu in 2003 and Fujian in 2011 [3], [8], [35]. In this study, E30 accounted for 27.4% of detected enteroviruses, further reflecting it is an important meningitis pathogen in mainland China. In the VP1 phylogenetic tree, Chinese strains were grouped into two exclusive clusters with no foreign strains. Frequent travel might increase importation of other E30 lineages. Although most Shandong strains from meningitis patients belonged to the same phylogenetic cluster (Cluster 1 in Fig. 1), the mean *p*-distance for these strains was 0.049, significantly higher than that of EV71 or CVB1 strains. Considering the evolution rate of 8.3×10^−3^ substitutions per site per year for E30 VP1 coding region [36], the high genetic divergence (up to 10.2%) of these strains in Cluster 1 represents a local circulation of E30 for more than 10 years.

Virus isolation method used in the present study provided convenience for further sequencing of VP1 entire coding region. However, the issue of sensitivity has to be addressed. Although most of the known human enteroviruses can be easily propagated in RD and HEp-2 cell lines, a few serotypes may not be recovered. Moreover, due to no specific enterovirus surveillance system and the issue of disagreement on sampling, the specimen (especially CSF) cannot be obtained for a certain proportion of cases. Only specimens from 437 cases were obtained in this study during 2006**–**2012. So, the surveillance data presented in this study only reflects part of reality and underestimates the real number of HEV associated aseptic meningitis.

## Conclusion

The present study describes the serotypes and molecular epidemiology of enteroviruses associated with aseptic meningitis in Shandong, China, and reveals that besides the traditional meningitis serotypes such as E30 and CVB1, EV71 and other coxsackie A viruses related meningitis is an important concern in mainland China. A specific surveillance system for human enteroviruses is needed to be established for comprehensively understanding the circulating serotypes associated with severe neurological diseases in China.
